# Phaeochromocytoma of Adrenal Gland with Paroxysmal Hypertension

**Published:** 1954

**Authors:** G. M. Colson

**Affiliations:** Late Senior Medical Registrar, Royal Devon and Exeter Hospital.


### PHAEOCHROMOCYTOMA OF ADRENAL GLAND WITH PAROXYSMAL HYPERTENSION.

BY

G. M. COLSON, B.M., M.R.C.P.

*Late Senior Medical Registrar, Royal Devon and Exeter Hospital.*

Phaeochromocytomata are tumours of chromaffin tissue usually in the adren[illegible] medulla or, less commonly, in sympathetic ganglion tissue. They produce var[illegible] ing proportions of adrenaline and noradrenaline and these pressor substanc[illegible] induce hypertension which may be either paroxysmal or sustained. Althoug[illegible] the clinical picture has been clarified in recent years cases are still liable to pa[illegible] unrecognized, especially if the possibility of the condition is not borne close[illegible] in mind. The present report describes a case in which there were typic[illegible] symptoms for four years before the correct diagnosis was made. It also illustrat[illegible] some points in management and the dramatic improvement which may follo[illegible] successful treatment.

### CASE REPORT

W. B. a foundry worker aged forty-six, was referred to the out-patient department [illegible] the Royal Devon and Exeter Hospital in March, 1952, on account of frontal headach[illegible] lethargy, constipation and loss of weight. These symptoms were first noted in 1946 whe[illegible] he was normal on physical examination. In 1948 he described his headaches as sever[illegible] and occurring in " attacks " of sudden onset whilst dressing, lasting ten to fifteen minut[illegible] and associated with profuse sweating. Again on examination he was normal and h[illegible] blood pressure 120/85. Subsequently all symptoms became more troublesome and b[illegible] 1952 his weight had fallen by two stones, constipation was severe and his " attacks " o[illegible] headache more frequent. On mild exertion he was liable to sweat profusely. To direc[illegible] questioning he admitted that his " attacks " were frequently associated with stooping t[illegible] lift heavy metal plates at work. He would suddenly develop an uneasy feeling in his che[illegible] and neck, an intense throbbing, frontal headache, giddiness, pounding of his hea[illegible] within the chest and, feeling extremely ill, his face drawn and pale and his body drenche[illegible] in sweat, he would retire to the lavatory to vomit for five or ten minutes when his attac[illegible] would pass and he was able to resume his work feeling perfectly well.

On examination he was thin, slightly pale, sweaty and had an anxious facial expressio[illegible] The cardiac rhythm was regular, rate 84 per minute, and the blood pressure 130/90. The heart was not clinically enlarged, the heart sounds were normal and the lung base[illegible] were dry. There were no abnormal masses palpable in the abdomen. The optic disc[illegible] were normal, the arteries slightly spastic, the veins overfilled, and there were scatter[illegible] deep haemorrhages and moderate exudates, particularly at the macula.

Towards the end of the examination a spontaneous " attack " was observed. The pul[illegible] rate increased to 120 per minute, the heart overacted and the carotid pulsations wer[illegible] strikingly large. The pupils dilated, the facial colour changed to a deathly white and swe[illegible] stood out on his forehead. The blood pressure was taken immediately and found to b[illegible] 290/160. During the next five minutes the blood pressure fell steadily to 125/95 b[illegible] which time he had regained his normal composure. A clinical diagnosis of phaeochromo[illegible] cytoma was made and the patient admitted for investigation.

During the next three weeks his temperature was frequently raised to 99° F. and th[illegible]

resting pulse fluctuated between 80 and 100 per minute. The blood pressure, recorded several times daily, remained at 125/95 with the exception of three mornings when the patient complained of headache and the blood pressure rose to 190/140.

The cold pressor response was normal. The response to changes of posture indicated lability of the blood pressure, but postural hypotension and tachycardia were not observed. An intravenous histamine test (Roth and Kvale, 1945) using 0·0125 mg. histamine acid phosphate was positive. From a resting basal blood pressure level of 130/95 there was an immediate fall to 110/80 followed by a dramatic rise in twenty seconds to 300/200 accompanied by the signs and symptoms identical with those of a spontaneous attack. Within seven minutes the blood pressure had returned to 125/90.

The blood count was normal. The blood urea was 58 mg. per 100 ml. The urea clearance (Van Slyke) was normal. Examination of the urine showed a light cloud of protein and the presence of a few hyaline and granular casts. The fasting blood sugar was normal. The heart was normal on screening. An X-ray of the abdomen showed no abnormal calcification. An intravenous pyelogram revealed considerable downward displacement of the left kidney with acute angulation of the ureter at the pelvi-ureteral junction: the right side was normal. An electrocardiogram showed the pattern of left ventricular strain.

A confident diagnosis of phaeochromocytoma of the left adrenal gland was made and on 23rd April operation was performed by Mr. K. P. S. Caldwell. Dr. J. Powell induced anaesthesia with pentothal sodium 0·6 g., followed by nitrous oxide, trichlorethylene and a minimal amount of ether, and continued by the endotracheal route. The patient having been placed in position, tubocurarine chloride 15 mg. was injected intravenously and a further 5 mg. administered subsequently. Through a left lumbar incision a tumour approximately 5 in. × 4 in. was found in the left adrenal gland and removed. The blood pressure remained steady at 120/80 until the tumour was manipulated but then rose suddenly to 280 systolic and finally became unrecordable whilst the vascular pedicle of the tumour was ligated. An intravenous injection of 18 mg. piperidyl-methyl-benzodioxane (933F) was given without any demonstrable reduction of blood pressure. No attempt was made to explore the right adrenal gland in view of the alarming hypertension. On return to the ward the patient's blood pressure fell rapidly to an unrecordable level, he was cyanosed, sweating and his pulse was of small volume. An intravenous infusion of l-noradrenaline (7 micrograms per min.) was effective in restoring the blood pressure to 100/70 and was continued for twenty-four hours when its withdrawal was followed by no fall of pressure.

The subsequent course was uneventful. The blood pressure rose to 130/90 and remained at this level. Protein and casts disappeared from the urine, the blood urea returned to normal and before the patient left hospital the intravenous histamine test was repeated and was negative. When seen in November, 1952, the patient was symptomless and had regained his normal weight. Since operation his bowels had acted regularly each day. On examination he was a fit-looking man with no abnormal signs, the blood pressure being 130/90 and the optic fundi normal. The electrocardiogram had reverted to a normal pattern.

The tumour was oval in shape measuring 12·5 cm. by 10 cm. by 6 cm. and weighed 680 grams. The histological appearances were typical of phaeochromocytoma (Dr. G. Stewart Smith). Biological assay (Dr. W. S. Peart) indicated that the tumour contained 1800 mg. of adrenaline and 1220 mg. of noradrenaline. A sample of urine collected for fourteen hours before operation showed an excretion of 600 micrograms of amine and the ratio of noradrenaline to adrenaline was 0·5 (Case G of Hamilton *et al.*, 1953).

### DISCUSSION

This case illustrates the features typical of hypertensive episodes referable to an underlying phaeochromocytoma which, in retrospect, must have been present for at least four years. Sudden attacks of short-lived over-anxiety, headache, palpitation, facial pallor, sweating, nausea and vomiting should always arouse suspicion. Additional symptoms include shortness of breath, numbness, tingling and coldness of the extremities and epigastric distress which may radiate into the praecordium or neck. In about half the cases there is an aura which is recognizable but difficult to describe (Calkins *et al.*, 1951). Careful interrogation will reveal a history of liability to excessive sweating between attacks in 90 per cent. of patients (Smithwick *et al.*, 1950). Constipation was a prominent symptom in the case described and it is noteworthy that it disappeared immediately after operation. This is an unusual feature which has not been recorded by other observers.

Physical examination between paroxysms is frequently negative but abnormal signs which may be encountered include an unduly moist skin, tremor, tachycardia, pupillary dilatation, weight loss, facial pallor, cardiac enlargement, retinopathy and a palpable abdominal mass. The temperature is commonly elevated to 99–100° F., traces of protein and sugar may be present constantly in the urine and showers of hyaline casts intermittently (Pincoffs, 1929.)

The case described possessed so many of these classical features that the clinical diagnosis presented no difficulty. Unfortunately not all cases are equally straightforward and may be confused with anxiety state, thyrotoxicosis, diabetes mellitus, chronic nephritis, migraine and cerebral tumour (MacKeith, 1944). Of these differential diagnoses the most important is anxiety state in emotionally labile subjects, and unless a paroxysm is observed the distinction may be extremely difficult. It is in these cases that every effort should be made to overcome such difficulty by day-to-day observation and further investigation.

An X-ray of the abdomen may reveal patchy calcification at the site of a tumour if of sufficiently long standing. Intravenous pyelography is normal if the tumour is small but downward displacement of the kidney may be demonstrated if a larger tumour arises from the adrenal gland. Presacral oxygen insufflation is theoretically more likely to be of greater value in outlining smaller tumours but in practice often fails to do so—as in the case recently reported by Ward *et al.* (1953), where the right adrenal outline was considered normal but at operation a phaeochromocytoma one inch in diameter was discovered.

Engel and Euler (1950) stressed the diagnostic value of an increased urinary output of adrenaline and noradrenaline in phaeochromocytoma and whenever there is doubt about the diagnosis a 24-hour sample of urine should be assayed. The normal 24-hour output of amine, 20–70 $\mu$g. will be exceeded when a phaeochromocytoma is present, and may reach extremely high levels. Hamilton *et al.* (1953) consider that the estimation of urinary adrenaline and noradrenaline offers the best available special method of diagnosis and point out that no false positive result has yet been obtained.

Of the tests designed to stimulate the release of pressor substances from the tumour so that an attack may be observed, probably the most reliable is the intravenous histamine test introduced in 1945 by Roth and Kvale. As Richardson (1951) points out, its use should be restricted to those cases with normal pressures when the intermittent nature of the symptoms has led to the suspicion that paroxysmal hypertension is their cause. The test is not without risk and a hypotensive blocking agent should be at hand whenever it is performed.

The diagnostic use of antagonistic drugs such as piperoxane or regitin (C733[illegible]) is, of course, confined to cases with sustained hypertension and affords no help in those where the blood pressure is normal between paroxysms.

It is not proposed to discuss the details of the surgical approach or the factors governing the choice of anaesthetic which have been carefully considered by Ward *et al.* (1953). In the present case the lack of response to benzodioxane and the immensity of the tumour are of interest and probably not unrelated. In so far as benzodioxane exerts its action in competing with adrenergic pressor substances it is not surprising that a dose of 18 mg. failed to compete successfully with the high blood concentration of pressor substances which must have been attained at the height of operative manipulation. No doubt a larger dose might have counteracted the alarming hypertension during operation but only at the expense of greater postoperative hypotensive collapse.

### SUMMARY

A case of phaeochromocytoma in a man aged forty-six with symptoms from paroxysmal hypertension is described. The tumour, which weighed 680 grams, was successfully removed at operation.

My thanks are due to Dr. Charles Seward for permission to publish this case and to those mentioned in the text who were concerned in its management.

### REFERENCES

Calkins, E., Dana, G. W., and Howard, J. E. (1951). *J. Amer. med. Ass.*, **145**, 880.

Engel, A., and Euler, U. S. von (1950). *Lancet*, **2**, 387.

Hamilton, M., Litchfield, J. W., Peart, W. S., and Sowry, G. S. C. (1953). *Brit. Heart. J.*, **15**, 241.

MacKeith, R. (1944). *Brit. Heart. J.*, **6**, 1.

Pincoffs, M. C. (1929). *Tr. Ass. Amer. Physicians*, **44**, 295.

Richardson, J. S. (1951). *Practitioner*, **166**, 556.

Roth, G. M., and Kvale, W. F. (1945). *J. Lab. clin. Med.*, **30**, 366.

Smithwick, R. H., Greer, W. E. R., Robertson, C. W., and Wilkins, R. W. (1950). *New. Engl. J. Med.*, **242**, 252.

Ward, G. E. S., Riches, E. W., and Johnson, B. (1952). *Arch. Middlesex Hosp.*, **2**, 133.

